# Breast cancer survivors’ recollection of their illness and therapy seven years after enrolment into a randomised controlled clinical trial

**DOI:** 10.1186/s12885-015-1573-6

**Published:** 2015-07-29

**Authors:** Patricia Lindberg, Michael Koller, Brunhilde Steinger, Wilfried Lorenz, Jeremy C. Wyatt, Elisabeth C. Inwald, Monika Klinkhammer-Schalke

**Affiliations:** 1 Tumor Center Regensburg e.V., An-Institute of the University of Regensburg, Regensburg, Germany; 2 Center for Clinical Trials, University Hospital Regensburg, Regensburg, Germany; 3 Leeds Institute of Health Sciences, University of Leeds, Leeds, UK; 4 Department of Gynecology and Obstetrics, University Medical Center Regensburg, Regensburg, Germany

**Keywords:** Breast cancer, Survivorship, Qualitative analysis, Quality of life, Patient-physician agreement, Complex intervention

## Abstract

**Background:**

Little is known about the subjective experience of breast cancer survivors after primary treatment. However, these experiences are important because they shape their communication about their illness in everyday life, usage and acceptance of healthcare, and expectations of new generations of patients. The present study investigated this topic by combining qualitative and quantitative methods.

**Methods:**

Breast cancer survivors in Bavaria, Germany were mailed a questionnaire up to seven years after enrolment into a randomised controlled clinical trial and start of their therapy. This enquired about their worst experiences during the breast cancer episode, positive aspects of the illness and any advice they would give to newly diagnosed patients. A category system for themes was systematically created and answers were categorised by two independent raters. Frequencies of key categories were then quantitatively analysed using descriptive statistics. In addition, local treating physicians gave their opinion on the response categories chosen by their patients.

**Results:**

133 (80 %) of 166 eligible patients who survived up to seven years returned the questionnaire. The most prominent worst experience reported by survivors was *psychological distress* (i.e. anxiety, uncertainty; prevalence 38 %) followed by *chemotherapy* (25 %), and *cancer diagnosis* (18 %). Positive aspects of the illness were reported by 48 % with the most frequent including *change in life priorities* (50 %) and s*ocial support* (22 %). The most frequent advice survivors gave was *fighting spirit* (i.e. think positive, never give up; prevalence 42 %). Overall, physicians’ estimates of the frequency of these responses corresponded well with survivors’ answers.

**Conclusions:**

Although physicians’ understanding of breast cancer patients was good, psychological distress and chemotherapy-related side effects were remembered as particularly burdensome by a substantial part of survivors. On the one hand, patients’ quality of life needs to be assessed repeatedly during medical follow-up to identify such specific complaints also including specific recommendations to the physician for targeted psychosocial and medical support. On the other hand the advices and positive aspects of the disease, reported by the survivors, can be used to promote positive ways of coping with the illness.

## Background

During the last years there has been growing interest regarding survivorship in breast cancer patients. One reason is the increasing number of long-term survivors due to improved screening and earlier treatment. A bulk of studies has investigated quality of life in long-term breast cancer survivors [1–5]. Overall it has been shown that patients recover from most impairments during the first year of illness [6–9] resulting in a long-term quality of life that is comparable to the general female population in most dimensions [1, 5]. Nonetheless, some persistent specific complaints have been identified in breast cancer survivors, such as arm symptoms, reduced sexual functioning, or fatigue [3, 5, 10]. Besides, fear of recurrence is often prevalent and negatively affects well-being [1, 10, 11]. A systematic review [12] of ten studies on breast cancer survivors’ quality of life concluded that good quality of life more than five years after diagnosis was associated with the absence of chemotherapy or comorbidities, high income, and sufficient levels of social support. However, many breast cancer survivors also experienced persistent specific complaints, such as arm symptoms or sexual problems. Furthermore, numerous more recent studies investigated various therapeutic approaches (stress reduction, exercise, counselling, spiritual therapy) for improvement of quality of life in randomised controlled trials [13–16].

These studies investigating long-term quality of life of cancer survivors predominantly use standardised measures. To date, there exists no established questionnaire that focuses specifically on the quality of life of survivors. Therefore, most studies use instruments initially developed for monitoring the course of diagnosis and treatment, such as EORTC QLQ-C30 [17] or FACT-G [18]. Although these instruments have their merits, it is possible that they omit aspects of the cancer experience that are important for survivors. Qualitative research is one way to resolve this, as this allows us to explore the survivor perspective and elicit a wider spectrum of answers than closed-ended quality of life questions. Thus issues can be detected that are omitted from standardised questionnaires [19]. This may elicit new hypotheses that can be analysed quantitatively. A good example of this approach is the study by Lauver et al. [20] that combined quantitative and qualitative measures to explore stressors after the end of primary therapies. By using open-ended qualitative questions they identified “dealing with uncertainties” as a stressor which would have been otherwise overlooked.

Another relatively unexplored field is cancer survivors’ personal evaluation of their illness course [21]. The meaning of the cancer experience to 58 long-term survivors was investigated by Foley et al. [22] using interviews more than five years post-diagnosis. They demonstrated that most survivors reported either little impact of cancer or even a positive long-term influence on their lives, such as more inner strength and a greater appreciation of life. This kind of personal growth was associated with a better quality of life.

The starting point of this research was the patient perspective which is communicated in their everyday life to family, friends, other patients, and physicians and might influence women’s attitude toward the illness as well as their use and acceptance of health services, support services and alternative therapies. More specifically, the aim of the present study was to examine the recollections of breast cancer survivors seven years after diagnosis regarding their (1) worst experiences during the illness, (2) potential positive aspects of the disease, and (3) the advice they would give to fellow patients. Another aim was to investigate if these responses of patients correspond with the opinion of their physicians regarding breast cancer survivors’ worst and most positive experiences and advices (4).

## Methods

### Sample

The study sample consisted of 200 female primary breast cancer patients who had participated in a randomised controlled clinical trial investigating the use of standardised quality of life diagnostics and related therapies to improve patients’ subjective recovery [23]. All participants had been surgically treated between 2004 and 2006 in one of five participating certified breast cancer centres in Bavaria, Germany. To achieve high external validity, the trial inclusion criteria had no restrictions regarding disease stage or age [24]. Details about the theoretical background, method, and results of this complex intervention have been previously described [23, 25–27].

Follow-up of survivors was conducted up to seven years after breast cancer diagnosis in August and December 2012 (mean time since surgery 84 months; range 73–93 months). The term “survivor” is here used as five year survival of the cancer diagnosis, a criterion commonly accepted in cancer statistics [28, 29]. Therefore we supposed that the chosen time point for follow-up was adequate to investigate the perspective of “real” long-term survivors.

### Design

A cross-sectional design was used for the present study. This study constitutes Part IV (long-term implementation) of a large scale complex intervention project [30] on the routine use of quality of life data in oncological practice. Part I [26], II [27], and III [23] have already been published. Ethical approval had been obtained from the local university ethics committee (University of Regensburg, 03/197) and patients had given their informed consent. In August 2012 all eligible women were mailed a package of questionnaires supplemented by a stamped return envelope and a cover letter informing them about content and aims of the study. Patients who did not respond within six weeks received one reminder by telephone. Those who could not be contacted by phone were mailed a reminder with the questionnaire package [31]. There were no financial or other incentives to respond.

### Measures/instruments

#### Demographic and clinical variables

In this survey the following data were collected: age, marital status, number of children, education level, and employment status. Prognostic stage, type of surgical procedure, and adjuvant/ neo-adjuvant therapy were obtained from the original record of the randomised trial.

#### Qualitative questionnaire

The survivor questionnaire consisted of one page with three qualitative, open-ended questions:

1. *“Which was the worst experience regarding your cancer disease?”*
2. *“Have there also been positive aspects according to the illness?”*
3. *“Which advice would you give newly diagnosed breast cancer patients to cope with the disease?”*

This questionnaire had been tested beforehand in a pilot survey with breast cancer survivors who were not part of the randomised trial cohort and thus did not take part in the present survivorship study. Twelve women with an earlier diagnosis of breast cancer participated in the pilot study (mean time since diagnosis: 55.5 months, range 11–84 months) with a mean age of 58 years (s.d. ± 7.5, range 45–69 years). Pilot participants evaluated the qualitative questions as clearly formulated and easy to understand. No woman perceived the content of the qualitative questionnaire as unpleasant. Only one participant noted that the question asking for an advice for newly diagnosed patients was difficult to answer. Overall, the qualitative questionnaire was highly accepted and revealed useful insights into their illness so there was no need for modification.

### Developing a category system for qualitative answers

**Fig. 1:**
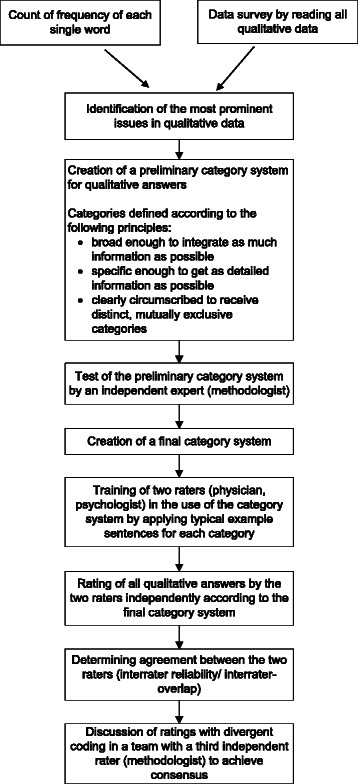
Sequence of each step in the qualitative data analysis process in the survivor study

To analyse the qualitative data, categories were generated by inductive analysis encompassing all prominent and relevant issues regarding the worst and positive experiences and advice for fellow patients (see Fig. 1). Because little is known about the investigated research field, candidate themes were derived from the data instead of using a predefined category system [32]. This was done independently by two investigators. Consensus was reached by discussion.

To quantify the proportion of responders mentioning each finding, answers were transferred to an electronic database (Microsoft Access 2010). In the first step, data were inspected using two different strategies: (1) using a word-by-word analysis, frequency of each word individually was counted using a computer program. Through this, issues which were commonly addressed by participants could be objectively identified, based on “Linguistic Inquiry and Word Count” (LIWC) by Pennebaker et al. [33]); (2) using a more holistic approach, the answers from all participants were read to become familiar with the data and their context. On the basis of (1) and (2) the most prominent issues in women’s answers were identified and transferred to a preliminary category system, considering the following criteria: each category should be broad enough to include as much information as possible, so that a reasonable picture of women’s answers could be given. At the same time categories were designed to be as specific as possible, to include meaningful information regarding content of the data. Furthermore, categories were clearly circumscribed and mutually exclusive so that data could be only assigned to one category.

The final category system contained 11 different categories for *worst experience*, 6 categories for *positive aspects* and 13 categories for *advice for fellow patients* (see Table 1). To facilitate the practical use of this coding system, each category was illustrated with a short description and text examples that resembled but were not identical to the patients’ original answers. Two raters (a physician and a psychologist) were trained in this category system and were then instructed to categorise all patient responses independently [34]. Some answers contained multiple statements belonging to more than one category. Those had to be divided into single issues and classified in different categories. Finally, both raters met with the third independent expert (methodologist) to discuss divergent ratings until consensus was achieved [35].

**Table 1:** Final category system used for the qualitative and quantitative analysis

Category	Description	Sample characteristic statements by the women
Worst experience		
Psychological distress	Anxiety or uncertainty about the course or outcome of the illness.	“uncertainty according to the outcome of surgery”; “uncertainty about the future”; “anxiety”; “fear of recurrence”; “fear of dying”
Chemotherapy	Chemotherapy or related side-effects.	“chemotherapy”; “loss of hair”; “to look at myself due to the loss of hair” “port implantation for chemotherapy”
Cancer diagnosis	The shock of receiving cancer diagnosis and the fact of being a cancer patient.	“communication of the diagnosis by the gynaecologist”; “Cancer itself! I have always lived a healthy life”; “that you have cancer and can’t forget it”
Mastectomy	Removal of the breast and the affected body image.	“removal of the breast, loss of self-esteem regarding sexuality”; “losing my breast or dying”; “disfigurement of the body, considerable restrictions in dressing”
Social burden	Fear of family or other conflicts in partnership or family caused by the illness.	“to have to be strong for my family”; “the fear of my twin sister and my daughter”; “that my husband couldn’t get along with the changes of my body, what I never had expected”; “to see, how my husband was suffering”;
Additional illnesses	Additional diseases like comorbidities or recurrence during or after breast cancer.	“I had a recurrence”; “cancer disease was accompanied by atrial fibrillations – bad health status for a long time”; “arm pain because my right upper arm was disabled by a fracture”
Radiotherapy	Radiotherapy with related side-effects.	“loss of energy because of radiation”; “after radiotherapy I had a pneumonia for nearly five years after treatment with cortisone”
Endocrine therapy	Endocrine therapy with related side-effects.	“endocrine therapy with all side effects”; “the obligation to take pills continuously despite circulatory complaints”; “my bones, probably affected by the intake of medication”
Nothing	No worst experience.	
Other		”pain”; “fatigue”; “that life will never be the same!”; “Everything happened at once. Diagnosis, divorce, driving test, moving house”
Positive aspects		
Change in life priorities	Change of one’s own priorities in life in terms of living life more consciously and relaxed, or changes in lifestyle.	“I think, you live more intensively and consciously”; “I have reconsidered my life, changed several things”; “I see a lot of things more relaxed”; “I have learnt to take more care of myself, to say ‘no’ more often that makes me proud”
Social support	Support by family, friends, or colleagues as well as unexpected help from others.	“the experience of intensive and also often unexpected support and friendship”; “my friends never abandoned me”; “I experienced a lot of attention, appreciation and support”; “I met wonderful people”
Good course of cancer	The good course and outcome of the illness.	“tumour was very small”; “no metastases”; “disease was early detected because of annual check-up”
Support by physicians/ nurses	The good (medical) treatment by physicians or nurses.	“the experience of caring physicians and nurses”; “advice and reassurance”; “the good medical attendance”
Gratitude	Being grateful to have survived.	“I have developed a profound feeling of gratitude”; “that I’m still alive”; “looking back on my life and thankfulness”; “regarding every day as a gift from God”
Other		“I could manage my disease very well”
Advice		
Fighting spirit	Think positive, fight, and never lose hope.	“never give up and think positive all the time”; “always thinking ‘Yes, I can manage that!’”; “Never lose hope!”
Information	Keep calm, get a second opinion, and inform yourself about the illness.	“inform yourself about all treatment options”; “don’t believe just one single physician”; “inform yourself intensively in the internet, get a second opinion”
Confidence in physicians	Trust your physician and follow his/ her instructions.	“do everything the doctor says”; “confidence in physicians”; “adherence to treatment”
Openness	Confide in somebody and talk a lot about the illness.	“positive conversations, share your experiences”; “talking a lot about the illness”; ““don’t hide the disease”
No advice	It is not possible to give any advice for fellow patients.	“I can’t give any advice”; “none, every patient comes to terms with it another way”
Business as usual	Don’t think too much about the illness, live life in a normal way.	“fade out disease of daily life, live for the moment”; “master everyday life as usual, domestic work, sports, friends, theatre”; “don’t think too much about disease, distract yourself, and remain cheerful”
Cancer screening	Have regular cancer screening.	“go to the doctor in time”; “regular cancer screening”; “early detection by screening”
Acceptance	Accept the illness.	“things you can’t change you have to accept”; “accepting disease”
Self-reflection	Reconsider your life.	“consider disease as a touchstone and if applicable as turning point, which is not solely negative but also offers opportunities to find oneself”; “attend to your own soul, find out, what makes you happy”
Belief in God	Strengthening in faith.	“pray a lot”; “don’t lose courage, my trust in God helped me a lot”
Support group	Visit a support group.	“contact other patients or a support group”; “visit a support group as soon as possible”
Secrecy	Keep your illness as a secret.	“Inform as few people as possible! Hardly anybody can help!”
Other		“Go to rehab”; “to undergo surgery immediately”; “no complementary medicine, take part in a trial”; “accept help”

### Survey of coordinating practitioners

Following completion of data analysis of the patient survey, a survey was conducted with those physicians who had taken care of the patients’ treatment and follow-up during the randomised trial [23]. Thus, the physicians were familiar with quality of life issues and would have managed one or more patients who were participating in the survivorship study. The aim of this survey was to compare physicians’ opinions regarding worst and most positive experiences of breast cancer survivors with the survivors’ actual perspective. A total of 50 eligible physicians were mailed a five-page questionnaire supplemented by a stamped return envelope and a letter explaining the study aim. The questionnaire referred to the three qualitative questions that were the focus of the patient survey (*worst experience*, *positive experience*, *advice to fellow patients*). After presenting the five most frequent categories from the patient survey for each of the three qualitative questions, the physicians’ task was to arrange the categories according to their expectation of the survivors’ response frequency, from “1” (most common answer) to “5” (less common answer). All the categories and category descriptions were taken from our analysis of the patient questionnaire data.

### Statistical analysis

Agreement between the two raters of categories was analysed using intercoder percent agreement and Cohen’s kappa to account for random agreement. Response categories were analysed quantitatively in a descriptive manner and reported as frequencies and proportions. For physicians’ ratings, means were calculated for each category as well as the percentage of each category ranked as number “1”. All data were analysed using SPSS software version 20.

## Results

### Participant characteristics

**Fig. 2:**
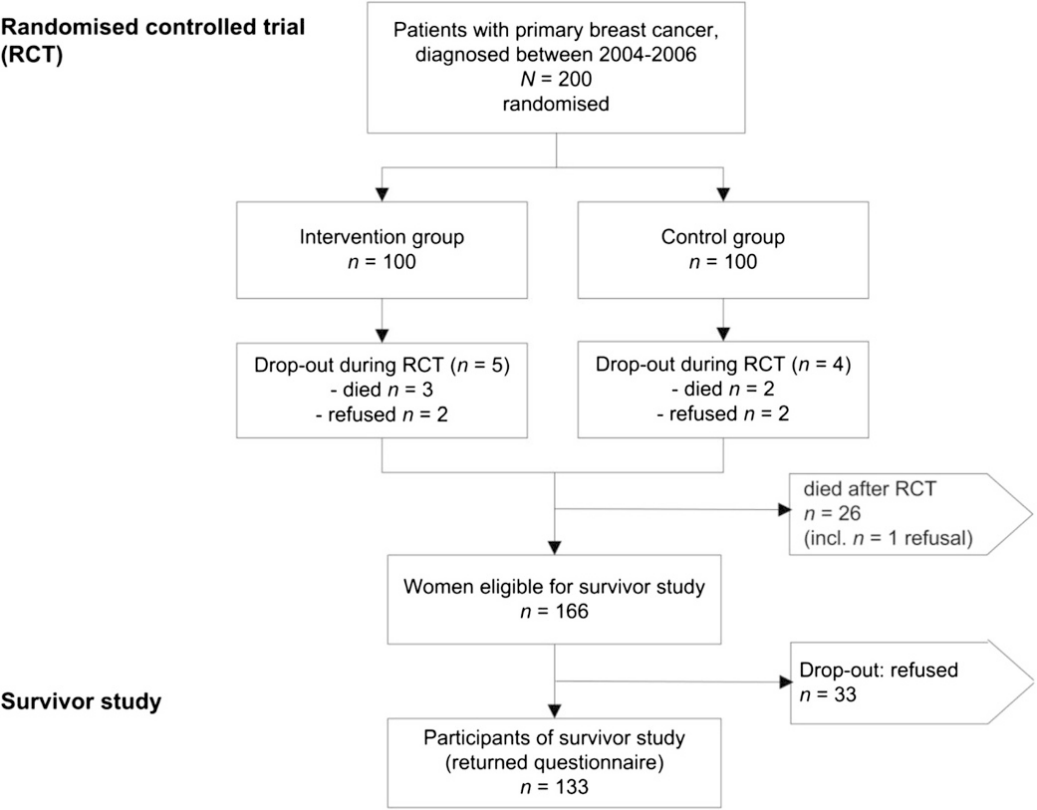
Patient recruitment in the survivor study. Breast cancer survivors, initially part of a randomised trial, enrolled onto long-term follow-up respecting drop-outs. Response rate of eligible patients in the survivor study 80 %

Of the 200 patients enrolled into the randomised study, three patients refused further participation and 31 had died at the time of the present study. Thus, 166 patients were eligible and 133 returned the questionnaire, a response rate of 80 % (Fig. 2).

The mean age of participants at long-term follow-up was 64.2 years (s.d. ± 10.8) and average interval since surgery was 84.8 months (s.d. ± 5.6). Further demographic and medical characteristics of participants are reported in Table 2.

**Table 2:** Demographic and medical characteristics of patient participants (*n* = 133)

		No. of patients	% of patients
Age (Mean ± s.d. 64.2 ± 10.8, range 41–92)			
	<50	8	6
	50–59	45	34
	60–69	36	27
	70–79	31	23
	80+	13	10
Months since surgery (Mean ± s.d. 84.8 ± 5.6, range 74–96)			
Marital status			
	Married	101	76
	Unmarried	4	3
	Divorced	14	11
	Widowed	14	11
Children			
	Children	113	85
	No children	10	8
	Unknown	10	8
Employment status			
	Employed full time	20	15
	Employed part time	27	20
	Retired/ not employed	80	60
	Unknown	4	3
Educational level			
	Did not finish school	2	2
	Compulsory	61	46
	Advanced vocational	56	50
	University	10	8
	Unknown	6	5
Cancer stage at diagnosis			
	UICC 0	2	2
	UICC I	68	52
	UICC II (II a and b)	39	29
	UICC III (III a, b, c)	21	16
	Unknown	3	2
Surgical procedure			
	Breast conserving therapy	106	80
	Mastectomy	27	20
Treatment (first year after surgery)			
	Chemotherapy	94	71
	Radiotherapy	118	89
	Endocrine therapy	113	85
	Anti-HER2 monoclonal antibody	11	8
Recurrent cancer		15	11

*s.d.* standard deviation

Respondents (*n* = 133) did not differ from non-respondents (*n* = 33) regarding age, time since surgery, stage, type of surgery, or recurrence of cancer.

133 respondents answered the questionnaire package including both the quantitative quality of life questionnaire (not reported in this paper) and the qualitative questions. Eight respondents missed this latter set of questions. Thus, qualitative analysis was based on 125 participating survivors.

### Qualitative analyses of worst and positive experiences and advice for fellow patients

#### Analysis of response length and word frequency

The length in words of participants’ responses was analysed: Women gave the longest answers when asked for *positive aspects* of their disease with a median of 10.0 words per answer (range 1–24 words), while responses describing the *worst experience* during cancer disease were shortest with a median of 5.0 words per answer (range 1–43 words). In between was *advice for fellow patients* (word length median 6.5, range 1–43 words).

In order to identify the most common issues in participants’ answers, frequency of each single word was counted electronically. The three most frequent nouns addressing *worst experience* were “anxiety” (*n* = 25), “chemotherapy” (*n* = 25), and “diagnosis” (*n* = 16). Asked for *positive aspects*, women most frequently used words like “life/ living” (*n* = 15), “illness” (*n* = 7), and “positive” (*n* = 7). When giving *advice for fellow patients*, the most frequent words were “positive” (*n* = 23), “illness” (*n* = 18), and “physician/s” (*n* = 12).

#### Interrater agreement

The median interrater percent agreement between the two raters was 98 % for categories regarding *worst experiences*, 93 % for *positive aspects*, and 99 % for *advice for fellow patients*. To account for random agreements Cohen’s kappa was also calculated. The median kappa was 0.83 for *worst experiences* (range 0.41–0.98), 0.75 for *positive aspects* (range 0.49–0.95), and 0.95 for *advice for fellow patients* (range 0.58–1.00) (it should be mentioned that for all three categories the lowest kappa was observed with respect to the response option “other”).

#### Worst experience regarding breast cancer

Of the 125 survivors answering the qualitative questionnaire, 118 (94 %) responded to the question asking for their *worst experience* during breast cancer (Table 3). By far the most prominent worst experience was *psychological distress*, reported by 38 %. The category included answers like “*uncertainty about the future”*, *“fear of recurrence”*, or *“fear of dying”*. This was followed by *chemotherapy* with 25 % (e.g. *“to look at myself due to the loss of hair”*, *“port implantation for chemotherapy”*), and *cancer diagnosis* with 18 % (e.g. *“communication of the diagnosis by the gynaecologist”*, *“Cancer itself! I have always lived a healthy life”*). The other defined categories were mentioned by less than 10 % of respondents. 12 % of issues were categorised as *other* including *“pain”* which was noted by only two survivors as one of their worst experiences (Table 3). Further examples of answers from individual patients are given in Table 1.

**Table 3:** Frequency of breast cancer survivors’ answers about their worst experience during breast cancer, positive aspects of the illness and advice for fellow patients

Worst experience (*n* = 118)	No. of patients	% of patients
Psychological distress	45	38
Chemotherapy	29	25
Cancer diagnosis	21	18
Mastectomy	9	8
Social burden	8	7
Additional illness	7	6
Radiotherapy	3	3
Endocrine therapy	3	3
Nothing	1	1
Other	14	12
Positive Aspects (*n* = 58)	No. of patients	% of patients
Change in life priorities	29	50
Social support	13	22
Good course of cancer	9	16
Support by physicians/ nurses	6	10
Gratitude	5	9
Other	3	5
Advice (*n* = 110)	No. of patients	% of patients
Fighting spirit	46	42
Information	17	16
Confidence in physicians	12	11
Openness	10	9
No advice	10	9
Business as usual	8	7
Cancer screening	7	6
Acceptance	6	6
Self-reflection	5	5
Belief in God	4	4
Support group	2	2
Discreteness	2	2
Other	9	8

#### Positive aspects of cancer

When asked if there had been also *positive aspects* of the disease, about half of the survivors affirmed this question (positive aspects: “yes” 60/125 (48 %); “no”: 54/125 (43 %); “missing” 11/125 (9 %)). Of those 60, 58 women gave written information about their most positive experience (Table 3). A *change in life priorities* was reported by 50 %. For example a woman answered *“I have reconsidered my life, changed several things”* and another described *“I have learnt to take more care of myself, to say ‘no’ more often that makes me proud”*. Furthermore 22 % of survivors mentioned the role of s*ocial support* by family, friends, and colleagues (e.g. *“the experience of intensive and also often unexpected friendship”, “my friends never abandoned me”*). 16 % named the *good course of cancer* (e.g. *“tumour was very small”, “no metastases”*) and 10 % emphasised (medical) *support by physicians and nurses* (e.g. *“the experience of caring physicians and nurses”, “the good medical attendance”*). The remaining categories *gratitude* and *other* were used by less than 10 % (see Tables 1 and 3).

#### Advice for fellow patients

This question was answered by 110 out of 125 women (88 %, Table 3). By far the most frequent advice for newly diagnosed patients was *fighting spirit* with 42 %. In this regard a woman recommended *“never give up and think positive all the time”* and another one advised *“Never lose hope!”*. Furthermore, 16 % of survivors suggested *information*, for example *“inform yourself intensively in the internet, get a second opinion”* or *“don’t believe just one single physician”*. Another 11 % advised *confidence in physicians*. (e.g. *“do everything the doctor says”*). Other categories were used by less than 10 % (see Tables 1 and 3).

### Evaluation of physicians

Of the 50 physicians contacted for the survivor survey, one was retired and could not be reached by mail. Of the remaining 49 doctors, 29 participated in the survey (59 %). Those had a mean age of 53.8 years (s.d. ± 9.0), were predominantly female (62 %) and all but one worked as gynecologist with length of professional experience in treating patients with breast cancer from 6 to 46 years (Median 23.5) (see Table 4). Four of the 29 participants did not fill in the questionnaire appropriately so their answers could not be analysed.

**Table 4:** Characteristics of participating physicians (*n* = 29)

		No. of physicians	*%* of physicians
Age (Mean ± s.d. 53.8 ± 9.0, range 35–72)			
	<40	1	4
	40–49	8	29
	50–59	12	43
	60–69	6	21
	70+	1	4
Sex			
	Female	18	62
	Male	11	38
Specialisation			
	Gynaecologist	28	97
	General practitioner	1	3
Professional experience			
	Breast cancer patients per year (Median 30.0, range 10–700)		
	<20	8	28
	20–50	13	45
	51–100	2	7
	101–199	4	14
	200+	2	7
	Years treating breast cancer patients (Median 23.5, range 6–46)		
	<10	2	7
	10–19	8	29
	20–29	11	39
	30–39	6	21
	40+	1	4

*s.d.* standard deviation

Overall, physicians’ estimates of the frequency of women’s experiences corresponded relatively well with their patients’ actual answers (Table 5). Regarding women’s *worst experience* during breast cancer, doctors and survivors named the same three issues most frequently (*cancer diagnosis*, *chemotherapy*, *psychological distress*). However, physicians underestimated the role of *psychological distress.* This was by far the most frequent answer of survivors (38 %), but was rated as the most common answer by only 16 % of physicians. Instead, 60 % of doctors thought that *cancer diagnosis* was the worst experience for patients (true answer 18 %).

**Table 5:** Physicians’ estimates of patients’ most frequent answers (*n* = 25)

	Physicians’responses		Patients’ responses
Worst experience	Mean	%	%^a^
Cancer diagnosis	1.8	60	18
Chemotherapy	2.5	24	25
Psychological distress	2.9	16	38
Social burden	3.4	-	7
Mastectomy	4.1	-	8
Positive aspects	Mean	%	%^a^
Change in life priorities	2.1	52	50
Social support	2.5	24	22
Good course of cancer	3.0	16	16
Gratitude	3.2	8	9
Support by physicians	4.1	-	10
Advice	Mean	%	%^a^
Fighting spirit	2.2	44	42
Openness	2.3	28	9
Information	2.8	21	16
Confidence in physicians/ nurses	3.3	8	11
Business as usual	4.1	4	7

^a^percentages based on raw counts of patients’ most frequent answers (multiple answers were possible, see Table 3); Mean = mean of ranks on a scale of 1–5; %: percentage of physicians rating the category as “most frequent answer” (response category “1” on a scale of 1–5)

Regarding *positive aspects* of cancer disease, physicians also showed a good correspondence with their patients, naming the issues in nearly the same order as the survivors (Table 5). They only underestimated the role of *support by physicians and nurses*. While 10 % of survivors reported this issue as their most positive experience during the illness, 52 % of doctors thought it would be the rarest answer given by patients.

When asked which *advice* breast cancer survivors might give to newly diagnosed patients, physicians (44 %) and survivors (42 %) similarly named *fighting spirit* most frequently. Seeking *information* about the illness was also frequently named by doctors (21 %) and women (16 %), while the role of *openness* was overestimated by physicians (28 %) compared with patients (9 %).

## Discussion

The subject of survivorship is of increasing interest due to the improved methods of cancer screening and therapy that prolong survival. The present study investigated how breast cancer patients remembered their illness episode about seven years after therapy onset. We are aware that such retrospective reports are vulnerable to distortions, such as recall and hindsight bias [36], reframing [37], and response shift [38, 39]. Different factors may contribute to recall bias [40] such as mood [41], the kind of material to be remembered (i.e. information in great detail) [42], or personal characteristics (i.e. optimism [43]). So, we deliberately bypassed the issue of “objectivity” and memory distortions, instead focusing on *subjective recollections* because these are the kinds of opinions and experiences that are communicated by survivors and shape the perceived stereotypes of breast cancer via their families, friends, and the media. These stories and stereotypes will in turn influence future generations of patients [21].

To our knowledge this is the only study investigating the perspective of breast cancer survivors regarding their course of illness which is also supplemented by the perspective of their physicians. The methodology we have used can act as a paradigm for others to investigate these issues. Open-ended, qualitative surveys go well beyond standardised assessment of quality of life and elicit new information. In contrast a recent study by Hollen et al. [44] investigated the importance of quality of life issues that are listed by breast cancer patients in general without assessing their subjective experiences in their course of treatment and recovery.

Before discussing our results, the strengths and limitations of the present study should be considered. The study population was well-defined due to patients’ participation in an earlier randomised trial [23]. The response rate was high given that the study was conducted more than six years after diagnosis (80 %). Furthermore, this investigation has high external validity (no exclusion according to age, stage, or recurrence, participants from both urban and rural areas). Further strengths are the emergent rather than predefined categories and the use of duplicate assessors to define the categories from the data.

However, there are also some limitations: First, we used a mailed survey instead of semi-structured interviews or focus group meetings. Therefore, most of the qualitative answers were short and enquiry for further explanation was not possible. The reason to choose this method anyhow was that our participants were already familiar with the questionnaire method. A mailed survey has also the advantage that there is less likelihood of social desirability than in interviews, no potential influence of the interviewer, and the accessibility of a larger sample of participants. Another limitation is the response rate of only 59 % in the physician survey. It is possible that only those doctors who have a particularly close relationship with their patients participated, so that the observed patient-physician agreement might be overestimated. Third, although this study can claim external validity for breast cancer patients in Germany, future studies need to confirm results in other countries and healthcare settings.

In qualitative research each category will contain a range of different perspectives. In order to address this problem we tried to be as objective and data-driven as possible when analysing patients’ qualitative statements. We therefore used word counting to identify objectively the most frequent issues in participants’ answers. In addition, two raters categorised the data independently. Nonetheless, we are aware that there is never a sole truth [45].

Keeping these pros and cons in mind, one key result is that the major part of our breast cancer survivors remembered *psychological distress* (such as fear of recurrence or uncertainty about the future) as their worst experience. A possible explanation might be the high prevalence of fear of recurrence which has been shown in long-term breast cancer survivors [11]. Thus, uncertainty and fear are still relevant to survivors so that these psychological complaints are also remembered as particularly burdensome during the illness. This information is directly relevant to physicians, who should anticipate psychological distress in their patients during the whole follow-up period and encourage patients to express their fears so that these can be discussed. Good patient-physician communication may help to reduce these fears and uncertainties and improve patient satisfaction [46] in order to prevent chronic psychological distress in long-term survivors [47].

In addition, *chemotherapy* was reported by one out of four women as particularly burdensome during the illness. These medical side effects have been already shown to be common psychosocial concerns in women with a recent diagnosis of breast cancer [48] and also remain one of the most burdensome experiences remembered by long-term survivors. Specific complaints of individual breast cancer patients need to get more attention during the time of medical treatment and follow-up. One solution to this problem is the regular assessment of the patient’s quality of life. These results need to be communicated to the physician with recommendations for tailored treatment of reduced quality of life [26]. This kind of intervention has been demonstrated to be effective for breast cancer patients during the first year of medical follow-up [23].

Another interesting result is that somatic symptoms seemed to play little or no role in the patients’ recollection of their illness. In particular, the concern over “pain” widely debated in oncology was almost never mentioned by patients in our survivor study. This information might help newly diagnosed breast cancer patients by taking away some of their fears.

Apart from this about half of the participants also reported positive effects related to the illness. This is notably lower than the percentage found by Sears et al. [49] with 83 % of breast cancer patients reporting at least one benefit in their disease. An explanation for these divergent results might be that Sears et al. surveyed recently diagnosed women whereas the present study focused on the perspective of long-term survivors. Perhaps finding benefit in the disease is especially important during the time of diagnosis as a form of coping. But in the long run it can be also maladaptive if expectations of benefits are not realised [50]. Similarly, Foley et al. [22] noticed that most cancer survivors experienced no impact of the disease on their lives.

Of those women in our sample who reported positive aspects most emphasised a change in life priorities. This is closely related to the concept of ‘*posttraumatic growth*’ - personally important changes as a result of a life-threatening crisis - often described in relation to cancer survivorship [51]. Similarly, a substantial number of survivors in our sample mentioned that they are living more intensively and consciously.

Furthermore, women evaluated the *social support* they received during the illness as a positive aspect. Although some survivors reported the *social burden* caused by cancer (fear of family members, conflicts in partnership) as their worst experience, a considerable number of the women experienced positive *social support* by family, friends, and colleagues. This seems to be an important aspect of coping with the illness, as it has been demonstrated that *social support* is a significant predictor for a better long-term quality of life in breast cancer patients [6]. Likewise, Sears et al. [49] identified the topic of social relationships as the most frequent benefit reported by recently diagnosed breast cancer patients. Similarly, *social support* was also relevant for survivors in the present study but not as important as a *change in life priorities*. Perhaps the meaning of positive experiences changes during the course of the disease. Whereas *social support* is most helpful in the acute phase of the illness its role becomes less important during survivorship. Instead, personal changes are more relevant because they are long-lasting and therefore can be still noticed as a benefit in long-term survivors. This needs to be investigated in future prospective studies.

*Support by physicians and nurses* was also stressed as a positive aspect during cancer. Medical staff need to be aware that they are an important aid for their patients in coping with breast cancer. This information relates to studies investigating patient-physician communication. Those identified that breast cancer patients perceive a caring attitude of the physician as more important than information-giving [52]. Likewise, physician attentiveness and empathy have been found to be associated with greater patient satisfaction and reduced emotional distress after the consultation in cancer patients [53]. In this sense the present study supports the assumed high relevance of patient-physician communication by demonstrating that a positive relationship with physicians and nurses is still important in the recollection of long-term survivors.

Survivors who reported positive aspects of their disease were also more likely to give advice to fellow patients compared to those women who did not remember any positive aspects. The vast majority recommended to think positive, to fight, and to never give up. This kind of *fighting spirit* has been found to be associated with better psychological adjustment to advanced breast cancer [54, 55]. Additionally, previous studies identified a positive relationship between trait optimism and wellbeing in breast cancer patients during the course of medical therapy [8, 56] and long-term follow-up [57]. The second most common advice was to inform oneself intensively about the illness e.g. by using the internet or by getting a second opinion. The specific information needs of cancer patients have been described in a systematic review including 112 articles published between 1980 and 2003 [58]. Reasons why some cancer patients do not want to get further information were qualitatively analysed by Leydon et al. [59] conducting in depth interviews with 17 cancer patients. They identified three motivations: hope (keeping away fearful, contradictory, or negative information to preserve hope), charity (concerns about taking up too much time of the doctor to the detriment of other patients), and faith (the doctor knows best, medical knowledge is too complex to understand). In agreement with the last explanation, some women in our sample adopted a passive posture by recommending *confidence in physicians* (do anything the doctor says, faith in physicians and medical therapy).

The study also demonstrated good but not perfect correspondence between physicians and patients. This is in contrast to the bulk of previous studies [60, 61]. There may be two explanations for this finding: (1) the measurement method, asking for direct experiences instead of standardised questionnaires and (2) the close relationship of our physicians with their patients as a result of regular follow-up, which might have been intensified by the randomised trial [62]. Physicians realised the affective dimension of the worst experience by choosing *cancer diagnosis* first (60 %) and *psychological distress* third (16 %). Patients had a similar opinion, although the two issues were reversed in frequency. Patients and physicians agreed that *psychological distress* and *cancer diagnosis* were particularly important elements of the cancer experience in the follow-up. The important lesson for physicians to learn is that distress caused by fear of recurrence or uncertainty about the future probably plays a more important role in the follow-up phase compared to the shock of diagnosis. So, in the medical encounter patients’ fears need to be addressed repeatedly, also discussing prognosis and the risk of recurrence. Another finding was that doctors tended to underestimate the important role they played for their patients: they need to be more aware of how much patients appreciate their medical and interpersonal support as an important aid in coping with their disease.

## Conclusions

This is the first study investigating the recollection of breast cancer survivors regarding their course of illness which is supplemented by the perspective of their physicians. Results demonstrated that physicians had a good overall understanding of the subjective experiences of breast cancer patients. Most survivors remembered psychological distress as their worst experience during breast cancer, followed by chemotherapy. These issues need to be considered even more in patient care. Physicians should address patients’ fears repeatedly in the medical encounter. Regular assessments of patients’ quality of life during medical follow-up can help to identify these specific complaints but should also include recommendations for the physician for targeted psychosocial and medical support. About half of the survivors also reported positive aspects of the illness, such as a change in life priorities. Their most frequent advice was fighting spirit. These advices and positive aspects of the disease should be further investigated to promote positive ways of coping with the illness.

Investigating the perspective of cancer survivors in this way is important since subjective recollections of former patients shape communication about the illness in everyday life, usage and acceptance of health care and ultimately, the expectations of new generations of patients.
